# Association between postoperative hypoalbuminemia and postoperative pulmonary imaging abnormalities patients undergoing craniotomy for brain tumors: a retrospective cohort study

**DOI:** 10.1038/s41598-021-00261-2

**Published:** 2022-01-07

**Authors:** Da-wei Zhao, Feng-chun Zhao, Xu-yang Zhang, Kai-yan Wei, Yi-bin Jiang, Dan Liu, Shui-xian Zhang, Hua Feng, Rong Hu

**Affiliations:** Department of Neurosurgery and Key Laboratory of Neurotrauma, Southwest Hospital, Third Military Medical University, Army Medical University, No. 30, Gaotanyan, Shapingba District, Chongqing, 400038 China

**Keywords:** Medical research, Risk factors

## Abstract

Hypoalbuminemia is associated with poor outcome in patients undergoing surgery intervention. The main aim for this study was to investigate the incidence and the risk factors of postoperative hypoalbuminemia and assessed the impact of postoperative hypoalbuminemia on complications in patients undergoing brain tumor surgery. This retrospective study included 372 consecutive patients who underwent brain tumors surgery from January 2017 to December 2019. The patients were divided into hypoalbuminemia (< 35 g/L) and non-hypoalbuminemia group (≥ 35 g/L) based on postoperative albumin levels. Logistic regression analyses were used to determine risk factors. Of the total 372 patients, 333 (89.5%) developed hypoalbuminemia after surgery. Hypoalbuminemia was associated with operation time (OR 1.011, P < 0.001), preoperative albumin (OR 0.864, P = 0.015) and peroperative globulin (OR 1.192, P = 0.004). Postoperative pulmonary imaging abnormalities had a higher incidence in patients with than without hypoalbuminemia (41.1% vs 23.1%, P = 0.029). The independent predictors of postoperative pulmonary imaging abnormalities were age (OR 1.053, P < 0.001), operation time (OR 1.003, P = 0.013) and lower postoperative albumin (OR 0.946, P = 0.018). Pulmonary imaging abnormalities [OR 19.862 (95% CI 2.546–154.936, P = 0.004)] was a novel independent predictors of postoperative pneumonia. Postoperative hypoalbuminemia has a higher incidence with the increase of operation time, and may be associated with postoperative complications in patients undergoing brain tumor surgery.

## Introduction

Hypoalbuminemia is associated with poor postoperative outcomes and complications in patients underwent surgical intervention, such as cardiac^1,2^, hand^3^, colorectal cancer surgery^4^ and orthopedic surgery^5,6^. In central nervous system, hypoalbuminemia is also associated with increased odds of mortality and a nonroutine hospital discharge^7,8^, and is a risk factor for postoperative acute kidney injury in patients who underwent craniotomy for tumor^9^. In addition, hypoalbuminemia is independent predictor of extended hospital stay following brain tumor surgery^10^.

Postoperative hypoalbuminemia has a considerable incidence in patients underwent craniotomy due to brain tumor in our institution. However, little is known about the association between preoperative clinical parameters and postoperative hypoalbuminemia in patients undergoing brain tumor surgery. Thus, the aim of our study was to investigate the incidence and the risk factors of postoperative albuminemia in patients underwent brain tumor surgery. Furthermore, we assessed the impact of postoperative hypoalbuminemia on postoperative complications after brain tumor surgery.

## Subjects and methods

### Patients

This retrospective study collected data from 372 consecutive adult patients who underwent craniotomy for brain tumors at the Department of Neurosurgery in the Southwest Hospital of the Third Military Medical University (Army Medical University) from January 2017 to December 2019. All study procedures and protocols involving human participants were in accordance with the ethical standards of the 1964 Helsinki Declaration, and the Ethics Committee of the First Affiliated Hospital of Army Medical University has approved this study (KY2021037). For retrospective study, formal consent is not required and the requirement for the informed consent of patients was waived by the Ethics Committee of the First Affiliated Hospital of Army Medical University prior to the collection of their medical data in this study.

### Inclusion and exclusion criteria

In this study, 372 adult patients with normal preoperative total (≥ 60 g/L) and albumin (≥ 35 g/L), who underwent craniotomy due to intracranial solid tumors, such as gliomas, meningiomas, acoustic neuromas, and metastatic tumors, were included. Patients who underwent craniotomy due to non-solid tumors, such as aneurysm, vascular malformation, intracerebral hemorrhage, hydrocephalus, cranioplasty or skull lesion, had been excluded. Patients younger than 18 years old, who had hypoalbuminemia (< 35 g/L) before operation were also excluded. Patients with abnormal proteinuria that may lead to protein loss were also excluded. For the purpose of this study, patients with postoperative serum albumin of < 35 g/L were defined as hypoalbuminemia group, while those with postoperative serum albumin of ≥ 35 g/L were defined as non-hypoalbuminemia group. The postoperative albumin level was checked on the first blood samples taken upon after craniotomy.

### Clinical data collection

For each patients, demographic data [age, sex, body mass index (BMI), histories of smoking and drinking], comorbidities (hypertension, diabetes mellitus, coronary heart disease, viral hepatitis, and history of stroke etc.), as well as pre- and post-operative laboratory test were collected. Operation time, intraoperative blood loss, intraoperative total input and intraoperative total urine output composed the operative-related medical data. Pathological classification were documented according to Central Nervous System Tumor Classification by the World Health Organization in 2016. The durations of postoperative neurological intensive care unit (NICU) and hospitalization and postoperative complications (pneumonia, pulmonary imaging abnormalities on the first postoperative chest CT image, epilepsy, incision infection, respiratory failure, renal failure, hydrocephalus, deep vein thrombosis) were also collected.

The pre- and first post-operative (within 6 h after surgery) laboratory tests included white blood cell count, red blood cell count, hemoglobin, hematocrit, platelet count, fibrinogen (FIB), prothrombin time (PT), prothrombin time-international normalized ratio (PT-INR), activated partial thromboplastin time (APTT), thrombin time (TT), D-dimers, potassium (K), sodium (Na), chloride (Cl), blood glucose, calcium (Ca), phosphorus (P), magnesium (Mg), serum prealbumin, total protein, albumin and albumin/globulin (A/G) ratio.

### Diagnosis criteria of pulmonary imaging abnormalities

Hydrostatic pneumonia is a type pulmonary infection, which usually results from chronic congestion and edema at bottom of lungs in critically ill patients who are bedridden in the long term due to cerebral apoplexy sequelae, cardiopulmonary failure and severe lung disease. Respiratory secretions are difficult to remove and become a good culture medium for bacteria resulting to pneumonia. Hypostatic pneumonia was diagnosed as follows: a white blood cell count > 12 × 10^9^/L or < 4 × 10^9^/L; presence of clinical symptoms, such as cough, fever, expectoration, and expectoration ability was weakened or disappeared; and a chest CT examination showed an irregular, patchy, highdensity shadow in the lower part of the lungs^11^.

In this study, pulmonary imaging abnormalities is defined as an abnormal imaging manifestation of lungs on the chest CT, which shows a patchy, crescent shaped, high-density shadow at the bottom of unilateral or bilateral lungs on the first postoperative chest CT, while there may not be typical clinical manifestation of pneumonia, such as cough, fever, dyspnea, gas exchange disorders, or does not meet the diagnostic criteria of pneumonia.

### Diagnosis criteria of pneumonia

The improved Centers for Disease Control and Prevention (CDC) criteria was used as diagnostic criteria for pneumonia in patients: (1) at least one of the following criteria: (a) fever (T > 38 °C) with no other recognized cause, (b) leukopenia (< 4 × 10^9^/L) or leukocytosis (> 12 × 10^9^/L), (c) age > 70 years, (d) altered mental status with no other recognized cause; (2) at least two of the following criteria: (a) new onset of purulent sputum, change in character of sputum over a 24 h period, increased respiratory secretions, or increased suctioning requirements, (b) new or worsening cough, dyspnea, or shortness of breath (respiratory rate > 25/min), (c) rales, crackles, or bronchial breath sounds, (d) gas exchange disorders (hypoxemia: PaO_2_/FiO_2_ ≤ 240; increased oxygen requirements). Diagnosis also required pulmonary CT appearance or progressive infiltration, consolidation, or ground glass^12^.

### Statistical analysis

All statistical analysis was performed using the SPSS software for Windows (version 25.0, IBM SPSS Inc., Chicago, IL) software. Descriptive statistical analysis of the data (e.g., means, medians, frequencies, and percentages) was performed. Normally distributed quantitative variables are presented as the mean ± standard deviation (SD) and were compared using independent unpaired two-tailed Student’s *t* test. Non-normally distributed quantitative variables are presented as the median [interquartile range (IQR)] and were compared using independent-sample nonparametric test. Categorical variables were expressed as counts with percentages and compared using the chi-squared test or continuity correction test. Logistics regression analysis was used to investigate the risk factors of postoperative hypoalbuminemia and pneumonia. The variables were analyzed by univariate binary logistic regression analysis. Multi-colinearity was assessed using the Pearson correlation coefficient statistic and by checking the Variance Inflation Factor multiple regression model with the same dependent and independent variables. Multivariate logistic regression model was performed on variables with significant differences (P < 0.05) determined in the univariate analysis to investigate the relationship between variables and postoperative hypoalbuminemia/pneumonia. All statistical tests of hypothesis performed at the 0.05 level of significance.

### Ethics approval

This study adheres to the principles of the Declaration of Helsinki. The study protocol was approved by the Ethics Committee of the Southwest Hospital of Third Military Medical University, China.

## Results

### Characteristics of patients underwent craniotomy for tumor

A total of 372 patients who underwent craniotomy for solid tumors [161 male (43.3%), 211 female (56.7%)] with a mean age of 48 ± 13 years (range 20–86 years) were enrolled. Of these, 333 (89.5%) patients developed hypoalbuminemia, whereas 39 (10.5%) patients remained normal albumin levels after craniotomy. There were significant statistical differences in BMI (P < 0.05) between with and without hypoalbuminemia patients. The patients with hypoalbuminemia had significant greater intraoperative blood loss, intraoperative total input, intraoperative total urine output, operation time and incidence of postoperative pulmonary imaging abnormalities than those without hypoalbuminemia after craniotomy (all P < 0.05). There were no significant differences in age, sex, previous diseases, lifestyle factors, pathological classification and other complications (all P > 0.05) between the two groups. The baseline characteristics of the two groups patients are shown in Table 1. There were significant statistical differences in preoperative laboratory tests, including red blood cells, PT, albumin, globulin, A/G ratio (P < 0.05) between with and without postoperative hypoalbuminemia patients (Table 2).

**Table 1:** Demographic and clinical characteristics between with and without hypoalbuminemia patients underwent craniotomy for brain tumor.

Variables	Non-hypoalbuminemia N = 39 (10.5%)	Hypoalbuminemia N = 333 (89.5%)	P value
Age (years, mean ± SD)	46 ± 13	48 ± 13	0.311
Sex [M, n (%)]	20 (51.3)	141 (42.3)	0.286
BMI	24.9 ± 2.7	23.4 ± 3.3	0.010
**Medical history [n (%)]**			
Hypertension	5 (12.8)	57 (17.1)	0.496
Diabetes	2 (5.1)	12 (3.6)	0.636
Coronary heart disease	2 (5.1)	5 (1.5)	0.115
Viral hepatitis	1 (2.6)	11 (3.3)	0.805
History of stroke	1 (2.6)	3 (0.9)	0.341
**Lifestyle factors [n (%)]**			
Smoking	12 (30.8)	82 (24.6)	0.403
Drinking	4 (10.3)	36 (10.8)	0.916
**Pathological classification [n (%)]**			
Meningiomas	13 (33.3)	131 (39.3)	0.288
Gliomas	9 (23.1)	111 (33.3)	
Acoustic neuromas	3 (7.7)	21 (6.3)	
Metastatic tumors	3 (7.7)	15 (4.5)	
Others	11 (28.2)	55 (16.5)	
Intraoperative blood loss [(mL, median (IQR)]	200 (200–300)	300 (300–500)	< 0.001
Intraoperative total input [(mL, median (IQR)]	2200 (1813–2688)	2850 (2300–3500)	< 0.001
Intraoperative total urine output [(mL, median (IQR)]	1775 (1400–2500)	2600 (2015–3350)	< 0.001
Operation time [(h, median (IQR)]	3.8 (2.7–5.0)	5.0 (4.0–6.0)	< 0.001
NICU time [(days, median (IQR)]	4 (3–5)	4 (3–5)	0.068
Hospitalization time [(days, median (IQR)]	21 (17–28)	21 (18–28)	0.524
**Postoperative complications [n (%)]**			
Pneumonia	–	18 (5.4)	0.137
Pulmonary imaging abnormalities	9 (23.1)	137 (41.1)	0.029
Epilepsy	3 (7.7)	10 (3.0)	0.131
Incision infection	–	3 (0.9)	0.552
Respiratory failure	–	11 (3.3)	0.249
Renal failure	–	3 (0.9)	0.552
Hydrocephalus	–	3 (0.9)	0.552
Deep vein thrombosis	–	2 (0.6)	0.627

*BMI* Body Mass Index, *NICU* Neurological Intensive Care Unit, *SD* standard deviation, *IQR* interquartile range.

**Table 2:** Comparison of preoperative laboratory findings between with and without hypoabluminemia patients underwent craniotomy for brain tumor (mean ± SD).

Variables	Non-hypoalbuminemia N = 39 (10.5%)	Hypoalbuminemia N = 333 (89.5%)	P value
White blood cells (10^9^/L)	6.13 ± 1.46	6.65 ± 2.45	0.198
Red blood cells, (10^12^/L)	4.79 ± 0.61	4.62 ± 0.48	0.046
Hemoglobin (g/L)	141 ± 17	137 ± 15	0.146
Hematocrit (%)	42.9 ± 4.8	41.9 ± 4.2	0.147
Platelets (10^9^/L)	216 ± 61	215 ± 61	0.933
FIB (g/L)	2.39 ± 0.48	2.57 ± 0.84	0.204
PT (s)	10.75 ± 0.52	11.00 ± 0.74	0.041
PT-INR	0.93 ± 0.05	0.95 ± 0.07	0.052
APTT (s)	27.33 ± 2.82	27.72 ± 3.73	0.523
TT (s)	17.07 ± 1.09	17.04 ± 1.17	0.896
D-dimer (mg/L)	0.23 ± 0.27	0.40 ± 1.14	0.352
K (mmol/L)	3.97 ± 0.39	4.03 ± 0.32	0.252
Na (mmol/L)	139.9 ± 2.4	140.0 ± 2.5	0.569
Cl (mmol/L)	105.5 ± 2.4	104.8 ± 2.9	0.160
Creatinine (μmol/L)	62.4 ± 13.9	64.2 ± 13.8	0.425
Glucose (mmol/L)	5.74 ± 1.29	5.71 ± 1.51	0.905
Ca (mmol/L)	2.40 ± 0.12	2.39 ± 0.19	0.763
P (mmol/L)	1.15 ± 0.17	1.20 ± 0.22	0.150
Mg (mmol/L)	0.88 ± 0.05	0.89 ± 0.08	0.656
Prealbumin (g/L)	0.26 ± 0.05	0.25 ± 0.06	0.221
Total protein (g/L)	72.3 ± 5.3	72.6 ± 5.6	0.743
Albumin (g/L)	44.3 ± 3.5	43.1 ± 3.6	0.036
Globulin (g/L)	27.9 ± 4.1	29.5 ± 4.0	0.019
Albumin/globulin ratio	1.62 ± 0.27	1.48 ± 0.22	< 0.001

*FIB* fibrinogen, *PT* prothrombin time, *PT-INR* prothrombin time-international normalized ratio, *APTT* activated partial thromboplastin time, *TT* thrombin time, *K* potassium, *Na* sodium, *Cl* chloride, *Ca* calcium, *P* phosphorus, *Mg* magnesium.

### Predictors associated with postoperative hypoalbuminemia

In univariate analysis, BMI, intraoperative blood loss, intraoperative total input, intraoperative total urine output, operation time, red blood cells, PT, albumin, globulin and A/G ratio were significantly associated with postoperative hypoalbuminemia (Table 3). The multivariate logistic regression analysis showed that operation time [OR 1.011 (95% CI 1.006–1.016, P < 0.001)], preoperative albumin [OR 0.864 (95% CI 0.768–0.972, P = 0.015)] and peroperative globulin [OR 1.192 (95% CI 1.058–1.344, P = 0.004)] were independent predictors of postoperative hypoalbuminemia for patients underwent craniotomy due to tumor (Table 4).

**Table 3:** Univariate logistic regression analysis of risk factors influencing postoperative hypoalbuminemia in the patients underwent craniotomy for brain tumor.

Variables	B	SE	Wald	P value	Odds ratio (95% CI)
BMI	− 0.141	0.056	6.354	0.012	0.869 (0.779–0.969)
Intraoperative blood loss	0.002	0.001	4.295	0.038	1.002 (1.000–1.004)
Intraoperative total input	0.001	0.000	15.191	< 0.001	1.001 (1.000–1.001)
Intraoperative total urine output	0.001	0.000	17.178	< 0.001	0.001 (1.001–1.002)
Operation time	0.008	0.002	16.073	0.000	1.009 (1.004–1.013)
Red blood cells	− 0.640	0.324	3.915	0.048	0.527 (0.280–0.994)
PT	0.613	0.292	4.424	0.035	1.846 (1.043–3.269)
Preoperative albumin	− 0.098	0.047	4.355	0.037	0.906 (0.826–0.994)
Preoperative globulin	0.109	0.047	5.470	0.019	1.115 (1.018–1.221)
Preoperative albumin/globulin ratio	− 2.400	0.700	11.761	0.001	0.091 (0.023–0.358)

*BMI* Body Mass Index, *B* regression coefficient, *SE* standard errors of regression coefficient, *PT* prothrombin time.

**Table 4:** Multivariate logistic regression analysis of risk factors influencing postoperative hypoalbuminemia in the patients underwent craniotomy for brain tumor.

Variables	B	SE	Wald	P value	Odds ratio (95% CI)
Operation time	0.001	0.003	18.118	< 0.001	1.011 (1.006–1.016)
Peroperative albumin	− 0.146	0.060	5.922	0.015	0.864 (0.768–0.972)
Peroperative globulin	0.176	0.061	8.294	0.004	1.192 (1.058–1.344)

Adjusted for body mass index, intraoperative blood loss, intraoperative total input, intraoperative total urine output, red blood cell, prothrombin time, preoperative albumin/globulin ratio.

*B* regression coefficient, *SE* standard errors of regression coefficient.

### Predictors associated with postoperative pulmonary imaging abnormalities

**Figure 1:**
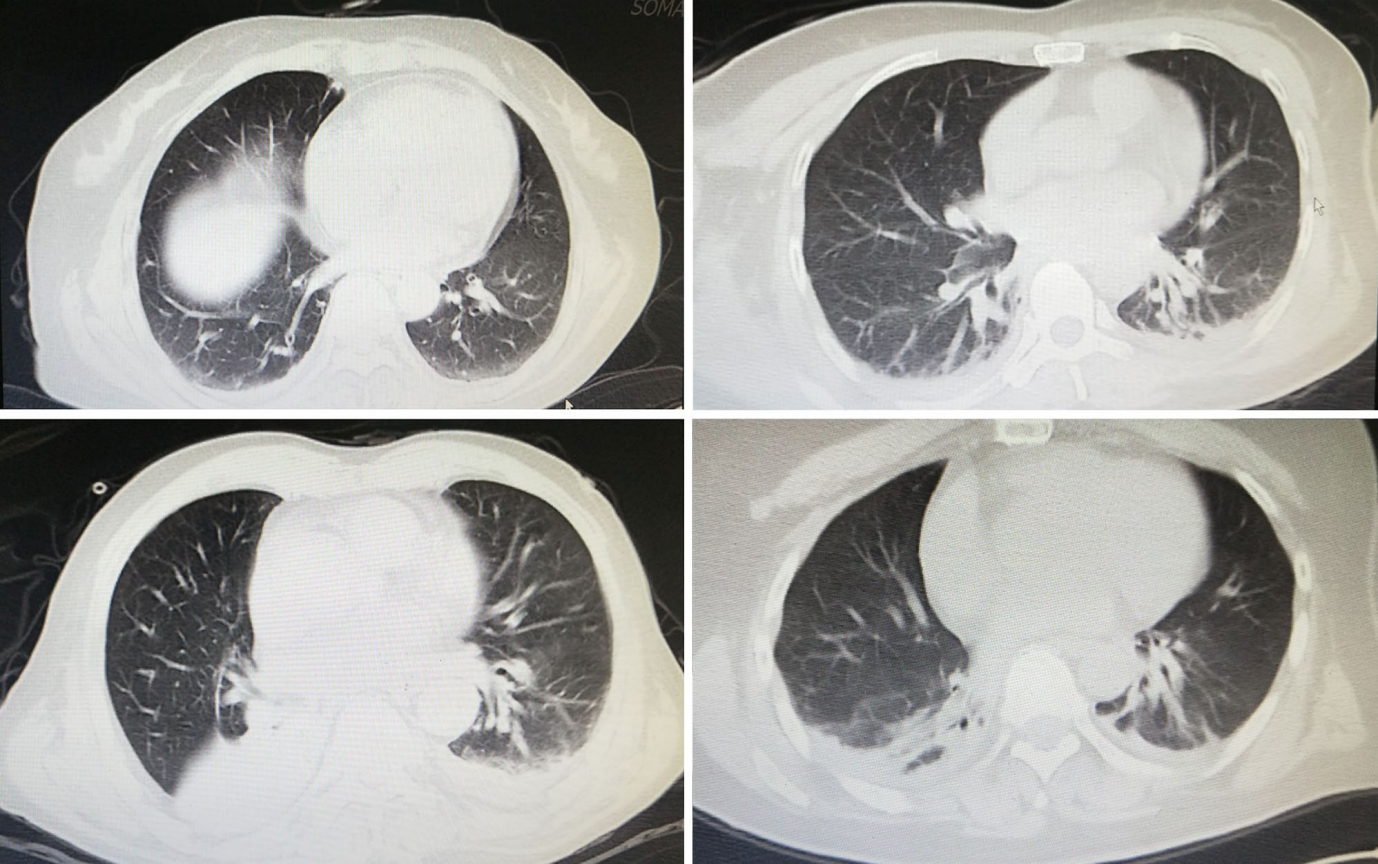
The first postoperative chest CT image showed bilateral pulmonary imaging abnormalities after craniotomy of brain tumor.

After craniotomy for brain tumor, the first chest CT imaging revealed 146 patients with bilateral pulmonary imaging abnormalities (Fig. 1). The overall incidence rate of postoperative pulmonary imaging abnormalities was 39.2% in total of 372 patients underwent craniotomy for brain tumor resection. Table 1 shows that the incidence of postoperative pulmonary imaging abnormalities in patients with hypoalbuminemia (41.1%) significantly higher than that in patients without hypoalbuminemia (23.1%) (P = 0.029). So we compared the baseline characteristics and postoperative laboratory test data of patients with and without postoperative pulmonary imaging abnormalities (Supplementary table 1), and introduced statistically significant variables into multivariate logistic regression analysis to explore the relevant risk factors of postoperative pulmonary imaging abnormalities.

The multivariate logistic regression analysis showed that age [OR 1.053 (95% CI 1.032–1.073, P < 0.001)], operation time [OR 1.003 (95% CI 1.001–1.005, P = 0.013)] and lower postoperative albumin [OR 0.946 (95% CI 0.903–0.990, P = 0.018)] were independent predictors of postoperative pulmonary imaging abnormalities for patients underwent craniotomy due to tumor (Table 5).

**Table 5:** Multivariate logistic regression analysis of risk factors influencing postoperative pulmonary imaging abnormalities in the patients underwent craniotomy for brain tumor.

Variables	B	SE	Wald	P value	Odds ratio (95% CI)
Age	0.051	0.010	27.100	< 0.001	1.053 (1.032–1.073)
Operation time	0.003	0.001	6.145	0.013	1.003 (1.001–1.005)
Postoperative albumin	− 0.056	0.024	5.631	0.018	0.946 (0.903–0.990)

Adjusted for age, diabetes mellitus, history of stroke, red blood cell, red blood cells, postoperative hemoglobin, postoperative hematocrit, postoperative fibrinogen, postoperative glucose, postoperative magnesium, postoperative prealbumin, postoperative total protein, postoperative albumin/globulin ratio.

*B* regression coefficient, *SE* standard errors of regression coefficient.

### Predictors associated with postoperative pneumonia

Of 372 cases, 18 patients eventually developed postoperative pneumonia, including 17 cases with and one case without pulmonary imaging abnormalities on the first postoperative chest CT image. The overall incidence rate of postoperative pneumonia 4.8% (18/372 cases). We compared the baseline characteristics and postoperative laboratory test data of patients with and without postoperative pneumonia (Supplementary table 2), and introduced statistically significant variables into multivariate logistic regression analysis to explore the relevant risk factors of postoperative pneumonia. The multivariate logistic regression analysis showed that coronary heart disease [OR 10.742 (95% CI 1.542–74.835, P = 0.017)], operation time [OR 1.005 (95% CI 1.000–1.010, P = 0.040], pulmonary imaging abnormalities [OR 19.862 (95% CI 2.546–154.936, P = 0.004)] and epilepsy [OR 8.371 (95% CI 1.942–36.075, P = 0.004)] were independent predictors of postoperative pneumonia for patients underwent craniotomy due to tumor (Table 6).

**Table 6:** Multivariate logistic regression analysis of risk factors influencing postoperative pneumonia in the patients underwent craniotomy for brain tumor.

Variables	B	SE	Wald	P value	Odds ratio (95% CI)
Coronary heart disease	2.374	0.990	5.747	0.017	10.742 (1.542–74.835)
Operation time	0.005	0.002	4.222	0.040	1.005 (1.000–1.010)
Pulmonary imaging abnormalities	2.989	1.048	8.132	0.004	19.862 (2.546–154.936)
Epilepsy	2.125	0.745	8.127	0.004	8.371 (1.942–36.075)

Adjusted for age, diabetes mellitus, postoperative albumin, operative time, intraoperative blood loss, renal failure.

*B* regression coefficient, *SE* standard errors of regression coefficient.

## Discussion

In this study, the overall incidence of postoperative hypoalbuminemia was 89.5% in brain tumor patients after craniotomy surgery. Patients with hypoalbuminemia had a higher intraoperative blood loss, intraoperative total input, intraoperative total urine output, operation time and incidence of postoperative pneumonia compared to the patients without hypoalbuminemia. Logistic regression analysis showed that operation time, preoperative albumin and peroperative globulin were independent risk factors of postoperative hypoalbuminemia in brain tumor postoperative patients. To our knowledge, there are few reports about the incidence and risk factors of postoperative hypoalbuminemia in patients after brain tumor surgery. In addition, the hypoalbuminemic patients showed a higher rate of postoperative pulmonary imaging abnormalities on the first postoperative chest CT image. Increasing age, extending operation time and decreasing postoperative albumin were risk factors of postoperative pulmonary imaging abnormalities. While coronary heart disease, operation time, pulmonary imaging abnormalities and epilepsy were independent predictors of postoperative pneumonia for patients underwent craniotomy for tumor.

For the mechanism of hypoalbuminemia, decreased intestinal absorption of protein due to poor oral intake, decreased synthesis of albumin due to hepatic dysfunction, increased catabolism of protein, albuminuria, and extensive vascular leakage of serum protein due to increased capillary permeability have been postulated^13^. In this study, the preoperative albumin of the patients were in the normal levels, and there was no significant differences between with and without postoperative hypoalbuminemia in the related diseases that may affect protein metabolism and loss, such as hepatitis and diabetes. In our institution, we performed a uniform preoperative fasting time for patients who prepared surgery for brain tumor. Therefore, we speculate that the early decrease of albumin may be related to the intraoperative blood loss and hemodilution caused by positive volume infusion^14^. While patients with hypoalbuminemia had lower levels in red blood cells, hemoglobin, hematocrit, platelets, total protein and A/G ratio (data not shown), which also proved that hemodilution may be responsible for dilution hypoalbuminemia. The extended operation time aggravates the occurrence of this process. The results proved that increasing operation time was an independent risk factor for postoperative hypoalbuminemia, which suggest that surgeons should shorten operation time as much as possible to avoid the risk of postoperative hypoalbuminemia and its related poor outcome.

Hypoalbuminemia is associated with poor outcomes in patients undergoing surgical intervention. In central nervous system, preoperative hypoalbuminemia affected the prognoses of patients with glioblastomas^15,16^. Hypoalbuminemia is also associated with increased odds of mortality and a nonroutine hospital discharge^7,8^, and is a risk factor for postoperative acute kidney injury in patients who underwent craniotomy for tumor^9^. In addition, hypoalbuminemia is independent predictor of extended hospital stay following brain tumor surgery^10^.

Postoperative pulmonary complications were significantly associated with poor outcome, including higher reoperation, readmission, mortality, and extended hospital stay in patients that surgical resection of brain tumors^17,18^. Previous study identified increasing operative time, increasing age, and increasing estimated blood loss, diabetes, chronic obstructive pulmonary disease, preoperative leukocytosis, American Society of Anesthesiologists classification ≥ 3, and infratentorial lesions as risk factors for postoperative pneumonia in postcraniotomy patients^17–19^. However, in infratentorial craniotomy with high risk for the development of postoperative pulmonary complications, the predictors for the occurrence of postoperative pulmonary complications were postoperative blood transfusion, lower cranial nerve palsy, prolonged ICU stay and tracheostomy^20^. Our result showed that pulmonary imaging abnormalities on the first postoperative chest CT image was more frequently incident in patients with hypoalbuminemia than in patients without hypoalbuminemia, with the incidence of 39.2%. We speculate that it may be mainly related to the respiratory tract management without sputum aspiration during long time operation. Increasing age, extending operative time and postoperative albumin were risk factors for postoperative pulmonary imaging abnormalities. To our knowledge, this is the first time to mention the incidence and risk factors of this imaging manifestation after craniotomy for tumor. Our results showed that the overall incidence of postoperative pneumonia was 4.8%, which is similar to the incidence of postoperative pneumonia of 1.3–3.11% reported in the literature^17,19^. However, there was no significant statistical difference in pneumonia between with and without hypoabluminemia patients. Heart disease^20^, prolonged operation time^17,19^, and epilepsy^21^ as risk factors for pneumonia were consistent with previous studies. Pulmonary imaging abnormalities provide a novel risk factor for pneumonia that warrants further investigation.

In addition, other postoperative complications, including epilepsy, incision infection, respiratory failure, renal failure, hydrocephalus and deep vein thrombosis, occurred in patients with hypoalbuminemia; while these complications did not occur in patients without hypoalbuminemia, except for 3 patients with epilepsy. At present, there is no obvious correlation between hypoalbuminemia and epilepsy, hydrocephalus, deep vein thrombosis. Hypoalbuminemia is a risk factor for surgical incision infection following various surgery^22–25^. Hypoalbuminemia is a risk for acute respiratory failure during hospitalization^26^ and postoperative acute kidney injury following craniotomy for tumor^9^.

We believe the results from our analysis can provide several benefits to neurosurgeons and neuroanesthesiologists alike. Most evident is the identification of brain tumor patients at risk for postoperative hypoalbuminemia, pulmonary imaging abnormalities and pneumonia, but perhaps more meaningful is the quantification of independent predictors. In addition, the results can provide several suggestions. Surgeons should shorten the operation time as much as possible, while neuroanesthesiologists should refine the management of intraoperative blood volume and respiratory tract.

Nevertheless, our present study also had several limitations. Firstly, this was a single-center retrospective case control study, which has obvious limitations. Secondly, whether preoperative albumin is normal or not as the inclusion criteria may lead to greater heterogeneity of patients. Thirdly, whether albumin supply and intensive respiratory tract management can improve the prognosis still needs to be verified by prospective randomized trials.

## Conclusions

The incidence of hypoalbuminemia after craniotomy for brain tumors was high, and the incidence of postoperative pulmonary imaginig abnormalities was higher in patients with hypoalbuminemia. The prolonged operation time was common independent risk factors for postoperative hypoalbuminemia, pulmonary imaging abnormalities and postoperative pneumonia. Moreover, our results demonstrate that hypoproteinemia have great potential as predictors of postoperative pulmonary imaging abnormalities, while pulmonary imaging abnormalities also is the risk factor of postoperative pneumonia. So we suggests that clinicians and neuroanesthesiologists need to provide early management of postoperative hypoproteinemia and intensive respiratory tract for the prevention of postoperative complications. However, a prospective study is needed to determine whether albumin supply and intensive respiratory tract management can improve the prognosis of patients underwent craniotomy due to brain tumor.

## Supplementary Information

Supplementary Tables.

## Data Availability

The datasets used and analysed to support the findings of current study are available from the corresponding author upon reasonable request.
