# Supplementary material for: Association between postoperative hypoalbuminemia and postoperative pulmonary imaging abnormalities patients undergoing craniotomy for brain tumors: a retrospective cohort study
Source: Sci Rep. 2022 Jan 7;12:64. doi: 10.1038/s41598-021-00261-2 (PMC8742077; doi:10.1038/s41598-021-00261-2)
Supplement: Supplementary file 1 — Supplementary Tables. [file 41598_2021_261_MOESM1_ESM.docx]

**Supplementary table 1. Comparison of parameters between with and without** **pulmonary imaging abnormalities patients underwent craniotomy for brain tumor (mean ± SD)**

| **Variables** | **Non-** **pulmonary imaging abnormalities**  **N = 226 (60.8%)** | **Pulmonary imaging abnormalities N = 146 (39.2%)** | **P value** |
| --- | --- | --- | --- |
| Age (years, mean ± SD) | 45 ± 13 | 52 ± 12 | <0.001 |
| Diabetes | 4 (1.8) | 10 (6.8) | 0.012 |
| History of stroke | - | 4 (2.7) | 0.012 |
| Operation time [(h, Median (IQR)] | 5.0 (3.7-5.8) | 5.1 (4.0-6.5) | 0.014 |
| **Postoperative laboratory tests** |  |  |  |
| Red blood cells, (10^12^/L) | 3.80 ± 0.64 | 3.59 ± 0.61 | 0.002 |
| Hemoglobin (g/L) | 113 ± 19 | 109 ± 17 | 0.049 |
| Hematocrit (%) | 33.8 ± 5.4 | 32.7 ± 5.0 | 0.041 |
| FIB (g/L) | 1.71 ± 0.63 | 1.86 ± 0.68 | 0.039 |
| Glucose (mmol/L) | 5.94 ± 1.60 | 6.34 ± 2.11 | 0.040 |
| Mg (mmol/L) | 0.78 ± 0.12 | 0.75 ± 0.09 | 0.014 |
| Prealbumin (g/L) | 0.19 ± 0.05 | 0.18 ± 0.04 | 0.019 |
| Total protein (g/L) | 53.3 ± 7.6 | 51.3 ± 7.7 | 0.015 |
| Albumin (g/L) | 29.1 ± 5.2 | 27.1 ± 5.3 | <0.001 |
| Albumin/globulin ratio | 1.22 ± 0.23 | 1.13 ± 0.25 | 0.001 |

FIB, fibrinogen; Mg, magnesium.

**Supplementary table 2. Comparison of parameters between with and without pneumonia patients underwent craniotomy for brain tumor (mean ± SD)**

| **Variables** | **Non-pneumonia**  **N = 354 (95.2%)** | **Pneumonia**  **N = 18 (4.8%)** | **P value** |
| --- | --- | --- | --- |
| Age (years, mean ± SD) | 48 ± 13 | 54 ± 14 | 0.036 |
| Sex [M, n (%)] | 152 (42.9) | 9 (50.0) | 0.555 |
| BMI | 23.6 ± 3.2 | 23.7 ± 3.3 | 0.906 |
| Medical history [n (%)] |  |  |  |
| Hypertension | 60 (16.9) | 2 (11.1) | 0.517 |
| Diabetes | 11 (3.1) | 3 (16.7) | 0.003 |
| Coronary heart disease | 5 (1.4) | 2 (11.1) | 0.003 |
| Viral hepatitis | 12 (3.4) | - | 0.427 |
| History of stroke | 4 (1.1) | - | 0.650 |
| Lifestyle factors [n (%)] |  |  |  |
| Smoking | 91 (25.7) | 3 (16.7) | 0.389 |
| Drinking | 39 (11.0) | 1 (5.6) | 0.466 |
| Intraoperative blood loss [(mL, Median (IQR)] | 300 (200-500) | 400 (275-525) | 0.326 |
| Intraoperative total input [(mL, Median (IQR)] | 2700 (2200-3450) | 3200 (2275-3880) | 0.360 |
| Intraoperative total urine output [(mL, Median (IQR)] | 2500 (2000-3200) | 2875 (2250-3600) | 0.432 |
| Operation time [(h, Median (IQR)] | 5.0 (3.8-5.9) | 5.5 (4.1-7.8) | 0.048 |
| NICU time [(days, Median (IQR)] | 4 (3-5) | 10.5 (6-15) | <0.001 |
| Hospitalization time [(days, Median (IQR)] | 21 (18-28) | 26 (15-49) | 0.283 |
| Postoperative complications [n (%)] |  |  |  |
| Pulmonary imaging abnormalities | 129 (36.4) | 17 (94.4) | <0.001 |
| Epilepsy | 9 (2.5) | 4 (22.2) | <0.001 |
| Incision infection | 2 (0.6) | 1 (5.6) | 0.021 |
| Renal failure | 2 (0.6) | 1 (5.6) | 0.021 |
| Deep vein thrombosis | 1 (0.3) | 1 (5.6) | 0.003 |
| **Postoperative laboratory tests** |  |  |  |
| Prealbumin (g/L) | 0.19 ± 0.05 | 0.18 ± 0.04 | 0.371 |
| Total protein (g/L) | 52.6 ± 7.7 | 52.1 ± 7.6 | 0.823 |
| Albumin (g/L) | 28.3 ± 5.4 | 27.7 ± 4.6 | 0.627 |
| Albumin/globulin ratio | 1.19 ± 0.24 | 1.17 ± 0.29 | 0.706 |
